# Supplementary material for: Allosteric regulation and crystallographic fragment screening of SARS-CoV-2 NSP15 endoribonuclease
Source: Nucleic Acids Res. 2023 Apr 28;51(10):5255–70. doi: 10.1093/nar/gkad314 (PMC10250223; doi:10.1093/nar/gkad314)
Supplement: gkad314_Supplemental_Files [file gkad314_supplemental_files.zip › Supplementary datasets.zip!/217/Refine_9/01-BUSTER/Cycle-2/shell.01/R_Rfree.html]

# Rxpct factors in resolution bins at cycle 0


|  |  |  |  |  |  |  |  |  |
| --- | --- | --- | --- | --- | --- | --- | --- | --- |
|  | **<d> (Å)**  Rxpct\_Work | Rxpct\_Free | Rxpct\_All | |  |  |  |  |
|  | 16.1098 | 0.3925 | 0.4442 | 0.3942 |  |
|  | 9.3010 | 0.2329 | 0.2790 | 0.2356 |  |
|  | 7.2045 | 0.1994 | 0.2614 | 0.2028 |  |
|  | 6.0889 | 0.2001 | 0.2680 | 0.2032 |  |
|  | 5.3699 | 0.1609 | 0.2458 | 0.1644 |  |
|  | 4.8573 | 0.1504 | 0.1937 | 0.1529 |  |
|  | 4.4681 | 0.1531 | 0.2119 | 0.1561 |  |
|  | 4.1595 | 0.1503 | 0.2262 | 0.1547 |  |
|  | 3.9072 | 0.1644 | 0.2397 | 0.1681 |  |
|  | 3.6958 | 0.1667 | 0.2277 | 0.1694 |  |
|  | 3.5155 | 0.1749 | 0.2134 | 0.1768 |  |
|  | 3.3591 | 0.1801 | 0.2340 | 0.1833 |  |
|  | 3.2220 | 0.1871 | 0.2248 | 0.1892 |  |
|  | 3.1003 | 0.1938 | 0.2093 | 0.1946 |  |
|  | 2.9915 | 0.1919 | 0.2382 | 0.1937 |  |
|  | 2.8934 | 0.2045 | 0.2292 | 0.2058 |  |
|  | 2.8044 | 0.2081 | 0.2327 | 0.2092 |  |
|  | 2.7231 | 0.2102 | 0.2693 | 0.2132 |  |
|  | 2.6484 | 0.2373 | 0.2890 | 0.2407 |  |
|  | 2.5796 | 0.2561 | 0.2889 | 0.2580 |  |
|  | **Overall** | 0.1892 | 0.2401 | 0.1917 |  |

# Rxpct factors in resolution bins at cycle 1


|  |  |  |  |  |  |  |  |  |
| --- | --- | --- | --- | --- | --- | --- | --- | --- |
|  | **<d> (Å)**  Rxpct\_Work | Rxpct\_Free | Rxpct\_All | |  |  |  |  |
|  | 16.1098 | 0.3930 | 0.4444 | 0.3947 |  |
|  | 9.3010 | 0.2327 | 0.2793 | 0.2355 |  |
|  | 7.2045 | 0.1998 | 0.2624 | 0.2032 |  |
|  | 6.0889 | 0.2004 | 0.2690 | 0.2035 |  |
|  | 5.3699 | 0.1609 | 0.2461 | 0.1644 |  |
|  | 4.8573 | 0.1503 | 0.1942 | 0.1529 |  |
|  | 4.4681 | 0.1531 | 0.2123 | 0.1561 |  |
|  | 4.1595 | 0.1502 | 0.2261 | 0.1546 |  |
|  | 3.9072 | 0.1643 | 0.2397 | 0.1680 |  |
|  | 3.6958 | 0.1666 | 0.2281 | 0.1693 |  |
|  | 3.5155 | 0.1748 | 0.2133 | 0.1767 |  |
|  | 3.3591 | 0.1800 | 0.2336 | 0.1831 |  |
|  | 3.2220 | 0.1871 | 0.2246 | 0.1892 |  |
|  | 3.1003 | 0.1937 | 0.2092 | 0.1945 |  |
|  | 2.9915 | 0.1918 | 0.2385 | 0.1936 |  |
|  | 2.8934 | 0.2045 | 0.2296 | 0.2058 |  |
|  | 2.8044 | 0.2081 | 0.2331 | 0.2092 |  |
|  | 2.7231 | 0.2102 | 0.2693 | 0.2132 |  |
|  | 2.6484 | 0.2368 | 0.2888 | 0.2402 |  |
|  | 2.5796 | 0.2557 | 0.2886 | 0.2576 |  |
|  | **Overall** | 0.1892 | 0.2403 | 0.1917 |  |

# Rxpct factors in resolution bins at cycle 2


|  |  |  |  |  |  |  |  |  |
| --- | --- | --- | --- | --- | --- | --- | --- | --- |
|  | **<d> (Å)**  Rxpct\_Work | Rxpct\_Free | Rxpct\_All | |  |  |  |  |
|  | 16.1098 | 0.3931 | 0.4445 | 0.3949 |  |
|  | 9.3010 | 0.2328 | 0.2795 | 0.2356 |  |
|  | 7.2045 | 0.1999 | 0.2626 | 0.2033 |  |
|  | 6.0889 | 0.2005 | 0.2691 | 0.2036 |  |
|  | 5.3699 | 0.1608 | 0.2460 | 0.1643 |  |
|  | 4.8573 | 0.1503 | 0.1944 | 0.1528 |  |
|  | 4.4681 | 0.1531 | 0.2126 | 0.1561 |  |
|  | 4.1595 | 0.1502 | 0.2260 | 0.1545 |  |
|  | 3.9072 | 0.1642 | 0.2397 | 0.1679 |  |
|  | 3.6958 | 0.1664 | 0.2283 | 0.1692 |  |
|  | 3.5155 | 0.1747 | 0.2132 | 0.1766 |  |
|  | 3.3591 | 0.1799 | 0.2334 | 0.1830 |  |
|  | 3.2220 | 0.1871 | 0.2245 | 0.1891 |  |
|  | 3.1003 | 0.1937 | 0.2092 | 0.1944 |  |
|  | 2.9915 | 0.1917 | 0.2388 | 0.1935 |  |
|  | 2.8934 | 0.2044 | 0.2297 | 0.2058 |  |
|  | 2.8044 | 0.2081 | 0.2332 | 0.2092 |  |
|  | 2.7231 | 0.2101 | 0.2693 | 0.2131 |  |
|  | 2.6484 | 0.2365 | 0.2889 | 0.2399 |  |
|  | 2.5796 | 0.2556 | 0.2878 | 0.2574 |  |
|  | **Overall** | 0.1891 | 0.2403 | 0.1917 |  |

# Rxpct factors in resolution bins at cycle 3


|  |  |  |  |  |  |  |  |  |
| --- | --- | --- | --- | --- | --- | --- | --- | --- |
|  | **<d> (Å)**  Rxpct\_Work | Rxpct\_Free | Rxpct\_All | |  |  |  |  |
|  | 16.1098 | 0.3894 | 0.4269 | 0.3907 |  |
|  | 9.3010 | 0.2327 | 0.2870 | 0.2359 |  |
|  | 7.2045 | 0.2005 | 0.2634 | 0.2039 |  |
|  | 6.0889 | 0.2043 | 0.2683 | 0.2072 |  |
|  | 5.3699 | 0.1615 | 0.2466 | 0.1650 |  |
|  | 4.8573 | 0.1499 | 0.1942 | 0.1525 |  |
|  | 4.4681 | 0.1508 | 0.2158 | 0.1541 |  |
|  | 4.1595 | 0.1498 | 0.2192 | 0.1538 |  |
|  | 3.9072 | 0.1604 | 0.2394 | 0.1643 |  |
|  | 3.6958 | 0.1627 | 0.2301 | 0.1657 |  |
|  | 3.5155 | 0.1707 | 0.2005 | 0.1721 |  |
|  | 3.3591 | 0.1759 | 0.2309 | 0.1791 |  |
|  | 3.2220 | 0.1863 | 0.2213 | 0.1883 |  |
|  | 3.1003 | 0.1929 | 0.2143 | 0.1939 |  |
|  | 2.9915 | 0.1921 | 0.2414 | 0.1940 |  |
|  | 2.8934 | 0.2057 | 0.2487 | 0.2079 |  |
|  | 2.8044 | 0.2084 | 0.2353 | 0.2096 |  |
|  | 2.7231 | 0.2094 | 0.2735 | 0.2126 |  |
|  | 2.6484 | 0.2371 | 0.3050 | 0.2415 |  |
|  | 2.5796 | 0.2566 | 0.2789 | 0.2579 |  |
|  | **Overall** | 0.1880 | 0.2404 | 0.1906 |  |
